# Identifying cisplatin-induced kidney damage in paediatric oncology patients

**DOI:** 10.1007/s00467-017-3765-6

**Published:** 2017-08-18

**Authors:** Chris D. Barton, Barry Pizer, Caroline Jones, Louise Oni, Munir Pirmohamed, Daniel B. Hawcutt

**Affiliations:** 1 Institute of Translational Medicine, University of Liverpool, Liverpool, UK; 2 Department of Paediatric Oncology, Alder Hey Children’s Hospital, Liverpool, UK; 3 Department of Paediatric Nephrology, Alder Hey Children’s Hospital, Liverpool, UK; 4 NIHR Alder Hey Clinical Research Facility, University of Liverpool, Liverpool, UK

**Keywords:** Cisplatin, Paediatrics, Oncology, Nephrotoxicity, Tubular toxicity, Magnesium

## Abstract

Cisplatin is one chemotherapeutic agent used to treat childhood cancer in numerous treatment protocols, including as a single agent. It is likely to remain in clinical use over the long term. However, cisplatin-related toxicities, including neurotoxicity and nephrotoxicity, are common, affecting treatment, day-to-day life and survival of such children. With one in 700 young adults having survived childhood cancer, patients who have completed chemotherapy that includes cisplatin can experience long-term morbidity due to treatment-related adverse reactions. A better understanding of these toxicities is essential to facilitate prevention, surveillance and management. This review article discusses the effect of cisplatin-induced nephrotoxicity (Cis-N) in children and considers the underlying mechanisms. We focus on clinical features and identification of Cis-N (e.g. investigations and biomarkers) and the importance of magnesium homeostasis and supplementation.

## Introduction

Improvements in the long-term survival of children with cancer in the UK means that approximately one in 700 young adults are survivors of childhood cancer [1]. However, these survivors experience long-term morbidity due to a variety of disease- and treatment-related adverse reactions that affect the quality of survival (QoS). Cisplatin is an essential chemotherapeutic agent used to treat childhood cancer, including osteosarcoma, neuroblastoma, hepatoblastoma, brain tumours and germ-cell tumours [2]. However, its use as either a single agent or in combination with other drugs induces specific toxicities, notably, neurotoxicity and nephrotoxicity. The closely related drug carboplatin is also used to treat childhood cancer, but its toxicity profile is clearly distinct from that of cisplatin, with an increased risk of myelotoxicity but with reduced nephrotoxicity in terms of both frequency and severity [3].

This review article discusses the phenomenon of cisplatin-induced nephrotoxicity (Cis-N) in children, including its mechanism of action in relation to renal injury and susceptibility factors, including clinical and genetic. We focus on clinical features and identification of Cis-N (e.g. investigations and biomarkers) and the importance of magnesium homeostasis and supplementation.

### Mechanism of action and pharmacology

Cisplatin covalently cross links the purine bases of DNA, interfering with DNA repair and inducing apoptosis through DNA damage, exerting its antitumour effects by a variety of well-described molecular mechanisms [4]. Tumour cells exhibit higher levels of oxidative stress in comparison with normal cells, with a greater production of reactive oxygen species (ROS) secondary to their increased metabolic activity, abnormal mitochondrial function and oncogenic pressures [4]. Cisplatin exposure further increases these levels of ROS, causing reduced glutathione, superoxide disumutase and catalase activity and subsequent loss of the key mechanisms required to scavenge ROS [4]. There is subsequently a loss of mitochondrial function arising from altered enzymatic function (e.g. phosphodiesterase), protein disturbance (e.g. loss of the protein sulfhydrl group) and loss of mitochondrial membrane potential [4]. The increased production of ROS in response to cisplatin exposure is related directly to both peak drug concentration and duration of drug exposure [5], leading to apoptosis and autophagy [4, 6, 7]. Cisplatin also exerts an anticellular effect through disruption of intracellular and mitochondrial calcium homeostasis, leading to lipid peroxidation, enzyme dysfunction (e.g. dehydrogenase inhibition secondary to glutathione depletion and general depletion of enzyme cofactors [4]), mitochondrial damage and inhibition and depletion of essential metabolites such as adenonsine triphosphate (ATP), contributing to cell apoptosis and necrosis [4]. Apoptosis is further induced through genotoxic stress arising from DNA cross linkage, direct activation of proapoptotic pathways [e.g. c-Jun N-terminal kinases (JNK)] [8] and activation of environmental stress pathways [e.g. mitogen-activated protein kinase (MAPK)] [9].

### Cisplatin-induced nephrotoxicity

Cisplatin toxicity arises most characteristically in the kidneys, cochlea, bone marrow, gastrointestinal mucosa and nerves (with altered taste, peripheral neuropathy and encephalopathy). The kidneys are particularly susceptible to cisplatin toxicity, as they are almost exclusively responsible for its excretion. The UK Renal Registry Report suggests that 1.9% of established all-cause end-stage kidney disease in children is secondary to malignancy, with 0.8% due to nephrotoxic drugs [10].

Small, heterogeneous populations from reported studies mean definitive data on the incidence and prevalence of renal injury in children receiving cisplatin is not conclusive. Most children will experience acute deterioration in renal function at some point in their treatment, but with considerable and unpredictable variations in both severity and reversibility [11, 12]. Tubulointerstitial injury is accepted as the most predominant form of Cis-N, with the concentration of cisplatin in the proximal renal tubular cells reported to be around five times higher than the peak serum concentrations [13]. The S3 segment of the proximal tubule accumulates the highest concentration of cisplatin, followed by the distal convoluted tubule and the S1 segment in the proximal tubule [14].

Renal clearance of cisplatin exceeds both creatinine clearance and glomerular filtration, indicating active secretion by the kidney, with an in vivo exponential plasma half-life of ∼30 min. While small amounts are present in bile and detectable in the intestinal lumen, faecal excretion is insignificant [15]. Numerous mechanisms for renal injury have been proposed (Tables 1 and 2). Renal uptake is by active transport, mediated by cell membrane transporter proteins, including (copper transporter-1 (CTR1) [4, 16] and organic cation transporter-2 (OCT2) [22, 23]. Several mechanisms for intracellular metabolism have been proposed, including conversion to cysteinyl–glycine conjugates and thiols and metabolism of cisplatin–glutathione conjugates by gamma glutamyl transpeptidase [17, 18].

**Table 1:** Mechanisms of cisplatin-induced kidney injury

Mechanisms of cisplatin induced kidney injury	References
Genotoxic stress from covalent cross-linkage DNA purine bases of DNA	[4, 13]
Apoptosis induced by DNA damage from impaired repair	[4, 6, 7, 13, 16]
Production of reactive oxygen species, increasing intracellular oxidative stress	[4, 13, 17, 18]
Disruption of intracellular and mitochondrial calcium homeostasis	[4, 13, 19]
Direct activation of proapoptotic pathways (e.g. JNK)	[8, 20, 21]
Activation of environmental stress pathways (e.g. MAPK)	[4, 9]
Inhibition of carnitine synthesis	[4, 19]
Focal susceptibility from pharmacodynamics (e.g. renal parenchymal accumulation/excretion)	[4, 16, 22–24]

*JNK* c-Jun N-terminal kinases,* MAPK* mitogen-activated protein kinase

**Table 2:** Factors that increase the risk of kidney injury in children receiving cisplatin

Risk factors for cisplatin-induced kidney injury	References
Increasing cumulative dose	[35]
Shorter administration time	[35]
Concurrent treatment with other nephrotoxins (e.g. ifosfamide/loop diuretics/aminoglycosides)	[36]
Increased peak serum/urine platinum concentrations (interindividual differences in pharmacokinetics)	[35, 39]
Increasing patient age	[41]

Accumulation of cisplatin within renal parenchymal cells occurs in a time- and concentration-dependent manner [24], with a progressive increase in the concentration of toxic moieties. Further kidney damage occurs secondary to the inhibition of carnitine synthesis and tubular reabsorption, further impairing mitochondrial function within the kidney [4, 19]. As renal tubular cells exist in an already predominantly hypoxic environment, synergy between these mechanisms of injury occurs.

Histone deacetylases (HDACS) are a group of enzymes that deacetylate specific lysine residues from both DNA-binding proteins (e.g. histones) and cellular-binding proteins [25], leading to the condensation of chromatin and general downregulation of gene expression [26]. HDAC inhibitors, e.g. valproate [27], sensitise tumour cells with synergistic effects on cisplatin cytotoxicity. With antiapoptotic and immunomodulatory activity demonstrated in kidney cells in vitro [25, 28, 29], HDAC involvement in cisplatin-mediated renal injury is likely, with HDAC-elicited pathways conferring renoprotection to cisplatin in animal models [25].

Magnesium is critical to cellular homeostasis and enzymatic reactions, including as the cofactor for ATP activity, mitochondrial respiration and nucleic acid and protein synthesis [30]. In the context of cisplatin administration, acute kidney injury (AKI) is enhanced by magnesium deficiency [31]. This is partly due to further stress on already disordered normal physiological processes. However, direct molecular mechanisms such as decreased renal expression of cisplatin efflux transporters in the presence of hypomagnesaemia have been elucidated in animal models (e.g. ATP-binding cassette subfamily C, member 6) [32]. AKI also arises secondary to the activation of other apoptotic pathways [33], incuding the linear chain ubiquitin assembly complex (LUBAC) [20] and necrotic/necroptotic pathway, such as receptor-interacting protein kinase 1 (RIP-1) [21].

### Drug-induced tubular injury: classification and relation to cisplatin

Recent literature describes a consensus classification of drug-induced kidney injury to bring consistency to its diagnosis and reporting [33]. Tubular dysfunction is characterised by renal tubular acidosis, Fanconi syndrome, syndrome of inappropriate antidiuretic hormone secretion (SIADH) through impaired water handling secondary to drug-related alterations in ADH secretion rather than direct tubular injury, diabetes insipidus and phosphate handling [33]. Primary criteria include one or more of the following: hypophosphataemia, glycosuria, hyperchloraemic metabolic acidosis and hypokalaemia or hyperkalaemia [33]. Secondary criteria include hypomagnesaemia, phosphaturia, hypouricaemia, tubular proteinuria and diabetes insipidus.

With regard to cisplatin, the mechanism of drug-induced injury is considered a type A reaction [34], which is dose dependent and predictable (in terms of mechanism if not extent of injury) and alleviated by dose reduction or drug withdrawal [33]. The time course is considered to be either subacute (occurring within 4 weeks and taking up to 90 days to resolve) or chronic (taking >90 days to resolve).

### Susceptibility to cisplatin-induced nephrotoxicity

Clinical, pharmacological and demographic risk factors for increased susceptibility to Cis-N have been clearly described, including increasing cumulative dose and administration time (e.g. 1 vs. 6 vs. 24 h, showing increased risk with short/intermittent boluses compared with prolonged infusions) [35], increasing patient age, and concurrent nephrotoxic agents (e.g. ifosfamide [36], loop diuretics and aminoglycoside antibiotics) (Table 1). Cisplatin pharmacokinetics are complex in vivo, affected by rapid plasma distribution, reversible and irreversible protein binding to both plasma and cellular proteins [37] and with considerable and unpredictable interindividual differences in cisplatin pharmacokinetics [37, 38]. The persistence of platinum moieties in the body up to 20 years after cessation of therapy may contribute to its long-term nephrotoxic effects [39].

Individual susceptibility factors predispose to different sites and mechanisms of injury. Renal injury correlates directly with peak serum and urine platinum concentrations [35, 40], whilst increasing age at treatment is associated with increased tubular toxicity and likelihood of hypomagnesaemia [40, 41]. Regardless, most children will experience an acute, unpredictable deterioration in renal function at some point during treatment [42].

Interindividual variability in predisposition to cisplatin-induced adverse effects is significant, with differences in toxicity greater than the variability seen in pharmacokinetics, despite equivalent doses. Genetic variants influencing regulation and expression of cisplatin metabolism and transport proteins (e.g. OCT2) have been described as risk factors for cisplatin nephrotoxicity [22, 23]. No study has yet undertaken a genome-wide approach. This contrasts cisplatin ototoxicity for which several studies (including genome-wide association studies) have been conducted to determine genetic predisposition [43].

### Preventing cisplatin-induced nephrotoxicity

In the paediatric setting, strategies are well established within chemotherapy protocols to minimise the risk of Cis-N. Patients receive posthydration infusions of cisplatin both before, during and after cisplatin administration as standard practice. They also receive slower cisplatin infusions (e.g. 24- vs. 1-h bolus), reducing peak serum levels of both cisplatin and toxic metabolites, promoting renal excretion, and reducing risk of Cis-N [35, 40]. Established drug regimens and current clinical trials include the use of formally measured glomerular filtration rate (GFR) [51-chromium-labelled ethylene diamine tetra-acetic acid (^51^Cr-EDTA)] at defined points of treatment, with reduction, delay or cessation of cisplatin treatment dependent upon the severity of any renal impairment.

Clinical pharmacokinetic studies previously indicated direct correlations between peak total and peak free plasma concentrations and renal toxicity [44–46]. Strategies such as extensive pre-/post-hydration and the use of mannitol [47] have become widely adopted in protocols, with the aim of driving enuresis and renal flow to remove excess and free cisplatin from vascular and renal spaces.

### Detectiing cisplatin-induced nephrotoxicity

Current modalities available for detecting Cis-N in the clinical setting are limited to structural or functional changes, including clinical parameters [48]. Changes in renal structures can be identified as histological or gross morphological, with histopathological assessment of kidney tissue the gold standard in the research setting (e.g. animal models). However, renal biopsy is rarely performed clinically.

Glomerular dysfunction may be manifest by elevated serum creatinine, albuminuria, tubular dysfunction by aminoaciduria, low molecular weight proteinuria, electrolyte loss and glycosuria, poor response to regulatory mechanisms (e.g. hypertension) and clinical effects of renal dysfunction (e.g. oliguria, requirement for electrolyte supplementation, growth restriction) [2, 49]). These can be monitored individually or in combination using validated clinical prediction tools [e.g. Acute Kidney Injury Network (AKIN), Pediatric Risk, Injury,Failure, Loss of Kidney Function, and End-stage KidneyDisease (pRIFLE), Clinical Practice Guideline for Glomerulonephritis (KDIGO) [50–52]).

Standard biomarkers rely on the measurement of serum electrolytes and markers of renal function. Serum creatinine is used as a marker of acute injury but its use is limited, as it typically rises late due to renal reserve [53]. It is also unreliable in patients with low muscle mass, which is often seen in children with cancer.

Thus, current biomarkers are inadequate for timely diagnosis of Cis-N, and there is a need for more sensitive detection methods in exposed children. As well as detecting transient, acute, tubulointerstitial renal injury—the most frequent clinical pattern of Cis-N—clinical investigations must also identify changes in glomerular and tubular function [37, 42, 51–55] and chronic disease courses [37, 42, 56–58]. Within paediatric oncology, changes in established markers of renal pathology have been investigated in patients receiving cisplatin. Increased levels of retinol-binding protein in the urine of paediatric patients receiving cisplatin suggest decreased physiological reabsorption indicative of tubular injury but do not provide information on prognosis [59]. Similarly, urinary albumin–creatinine ratio, urinary beta-2 microglobulin [54, 60] and assessments of phosphate excretion and tubular reabsorption have been used as measures of tubular injury [54, 61, 62]. Novel biomarkers such as kidney injury molecule-1 (KIM-1 [61]) and neutrophil-gelatinase-associated lipocalin (NGAL [62]) demonstrate potential clinical utility, with significant increases in levels following administration of cisplatin in human studies (Table 3 [59, 61–65]). However, neither novel nor more established markers of renal injury have been clinically validated and integrated into routine paediatric practice.

**Table 3:** Novel markers of cisplatin-induced nephrology (Cis-N) in children with cancer

Biomarker	Year	Sample (*N*)	Summary	References
Kidney-injury molecule-1	2015	22	Significantly increased levels of KIM-1 in adult patients receiving cisplatin	[63]
Kidney-injury molecule-1	2015	39	×2 and ×4 elevation in urinary KIM-1 levels at days 3 and 10 postcisplatin	[61]
Cystatin C	2008	22	18% increase in cystatin C serum levels after application of cisplatin	[64]
Neutrophil gelastinase-associated lipocalin	2013	33	Significant elevation of NGAL between 12 h and 4 days following administration of cisplatin	[62]

*KIM-1* kidney injury molecule-1,* NGAL* neutrophil-gelatinase-associated lipocalin

### Clinical implications of cisplatin-induced nephrotoxicity: hypomagnesaemia

Tubular damage represents an adverse, and often permanent, sequela of cisplatin exposure, with hypomagnesaemia the most common manifestation in both acute and chronic proximal tubular injury [63] at a prevalence between 30 and 100% depending on the timing of investigation [2, 11, 66–69]. This arises from magnesuria [2, 66, 70] and as part of the process of polyuria and deranged renal calcium metabolism and metabolic alkalosis in distal tubular toxicity [2]. Rat models have established specific molecular pathways: epidermal growth factor/transient receptor potential M6 (EGF/TRPM6) are downregulated in response to cisplatin exposure, leading to tubular magnesium loss [71], initiating further molecular mechanisms, including interactions between claudin-16 and -19 (known to influence tight membrane permeability) [72]. It is important to note, however, that hypomagnesaemia in this population of patients can be confounded by other factors, such as vomiting and gastrointestinal disturbance, making it difficult to determine specific causality.

Whilst predominantly an intracellular cation, it is extracellular magnesium concentrations that account for most signs and symptoms [71, 73–75], as follows:

- Muscle weakness/Cramps/Tetany/Twitching
- Constipation/Nausea/Vomiting/Loss of appetite
- Sensory loss/Numbness/Parasthesia/Tingling
- Headache
- Vertigo/Apathy/Depression/Fatigue/Anxiety/Insomnia
- Prolonged QT/Cardiac arrhythmias/Cardiac arrest
- Asthma/Wheeze
- Ataxia
- Cortical blindness/Seizures/Coma

When concurrent with other electrolyte imbalances, severe hypomagnesaemia can contribute to ventricular arrhythmias [74, 75]. Magnesium administered parenteral;y or intravenously aims at preventing the development of symptoms. Given established paediatric reference ranges for serum magnesium, the aim of supplementation is to normalise serum concentrations

**Fig. 1:**
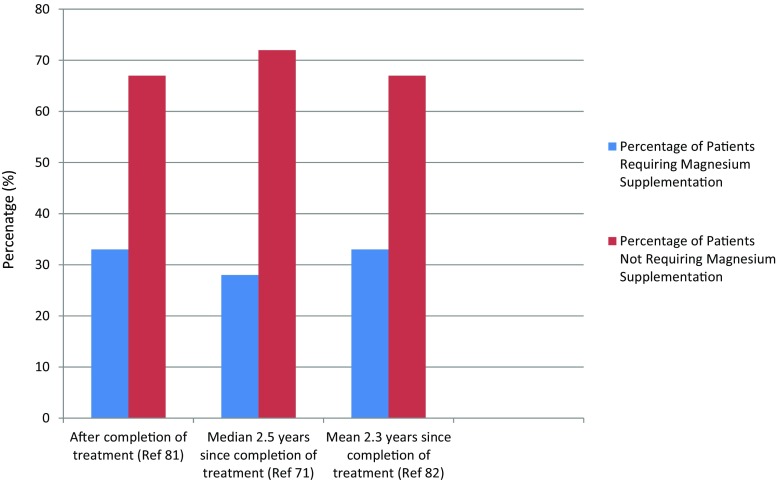
Paediatric oncology patients requiring magnesium supplementation (IV or orally) after completion of treatment with cisplatin; comparative data from three studies

Based on our own experience [76] and extensive review of the literature (unpublished data), hypomagnesaemia is amongst the most frequent manifestations of Cis-N. Sixty-eight patients from our single, primary paediatric oncology treatment centre were identified as having received cisplatin over a 10-year period (2001-2011). Data on the need for either oral or intravenous administration of magnesium supplementation prior to the end of treatment, as well as afterwards, was collated from electronic and pharmacy data, which was available for 65 patients, none of whom had a history of renal problems predating their diagnosis of cancer. Of those 65, 33% required magnesium supplementation (oral or intravenous) during treatment, with 27% requiring further supplementation after the completion of treatment [77] (Fig. 1). This is in keeping with other series, with ∼30% of patients having long-term hypomagnesaemia potentially requiring supplementation, e.g. 6/21 children at median follow-up of 2.5 years [11] and 6/18 at a mean follow-up of 2.3 years [77].

Although it is generally accepted that magnesium levels should be measured prior to cisplatin chemotherapy, there are no widely accepted or adopted guidelines for either overall monitoring of serum magnesium levels or treatment of hypomagnesaemia in children receiving cisplatin. It is important to note that while serum hypomagnesaemia reflects circulating magnesium depletion, only ∼1% of total body magnesium is present in serum [78]. This is despite evidence from both animal and clinical models that hypomagnesaemia may not only contribute to Cis-N but also that preventing hypomagnesaemia may even confer a degree of protection against cisplatin’s nephrotoxic effects. In a retrospective cohort of adult cancer patients receiving cisplatin, those who received magnesium supplementation IV experienced a statistically significant reduction in nephrotoxicity (6%) compared with those who did not (37%) [79]. Further, pretreatment magnesium supplementation in adult lung cancer patients has been demonstrated to reduce Cis-N [78], though specific mechanisms for this renoprotective effect are poorly understood.

Other rare but significant renal manifestations of Cis-N include chronic renal failure, Fanconi’s syndrome (during and after treatment [80]) and renal salt-wasting syndrome (RSWS). RSWS describes a very rare clinical syndrome of polyuria, volume depletion and loss of renal salts [81, 82], with only five of 23 case reports published since 1984 describing paediatric cases [81]. While the long-term sequelae of Cis-N include longitudinal growth restriction [83], the role of hypomagnesaemia, either acute or chronic, within this purview is not understood.

### Future perspectives

Further research is necessary to develop and validate phenotypes, assays or techniques to identify Cis-N in children. No standard methods of detection or classification exist for use in the clinical or research environment. While novel proximal tubular injury markers show promise for both more timely identification of Cis-N and more specifically delineating the site of kidney injury (e.g. KIM-1, NGAL), standardised clinical markers are needed for research to commence in this area.

Registries that identify patients from large numbers of centres will be needed for rare phenotypes. This will require standardisation of the phenotype both nationally and internationally. An alternative strategy would be to use magnesium depletion and supplementation required as a phenotype of tubular injury in children who have received cisplatin. This would have several advantages, including simplicity and objectivity. Additional work will be required to establish whether magnesium supplementation is a reliable and accurate marker of tubular toxicity. If proven, the routine clinical recording of the administration of medications (including supplements) to patients would allow this marker to be applied retrospectively, allowing retrospective data collection and increasing the rate of recruitment for pharmacogenomic and other studies of these uncommon conditions. In addition, further research is needed in the form of longer-term pharmacovigilance to identify risk factors for both clinical and subclinical chemotherapy-related renal impairment (e.g. delayed bone growth [84] and impaired growth and development [83, 84]).

## Conclusion

Cisplatin is an important drug for treating children with cancer. It is established in numerous treatment protocols, including as a single agent. As such, despite well-characterised adverse reactions, cisplatin will remain a first-line anticancer treatment for the foreseeable future, with no imminent alternatives with similar efficacy. CIs-N is likely to be underestimated in terms of both prevalence and severity, with implications for long-term effects and outcomes. While current clinical and biochemical biomarkers of kidney injury exist, none are standardised or validated relevant to the severity of, recovery from or long-term prognosis of Cis-N. The need for magnesium replacement, its route of administration and quantity and frequency holds potential utility both as research and clinical biomarkers for studying Cis-N in children with cancer.
